# Supplementary material for: Characterizing co-expression networks underpinning maize stalk rot virulence in Fusarium verticillioides through computational subnetwork module analyses
Source: Sci Rep. 2018 May 29;8:8310. doi: 10.1038/s41598-018-26505-2 (PMC5974142; doi:10.1038/s41598-018-26505-2)
Supplement: Supplementary file 1 — Supplementary Information [file 41598_2018_26505_MOESM1_ESM.docx]

***Supplementary Information***

**Characterizing co-expression networks underpinning maize stalk rot virulence in *Fusarium verticillioides* through computational subnetwork module analyses**

Man S. Kim^1,2*^, Huan Zhang^1*^, Huijuan Yan^1^, Byung-Jun Yoon^2^, and Won Bo Shim^1^

^1^ Department of Plant Pathology and Microbiology, Texas A&M University, College Station, TX 77843

^2^ Department Electrical and Computer Engineering, Texas A&M University, College Station, TX 77843

1. **Supplementary Materials and Methods**

**Preprocessing & subnetwork module prediction adopted from our previous approach**

Once *F. verticillioides*-maize stalk RNA samples (wild type and Δfsr1 mutant) were prepared, next generation sequencing (NGS) was performed using Illumina HiSeq 2000. Sequence cluster identification, quality prefiltering, base calling, and uncertainty assessment were also performed in real time using Illumina's HCS 1.5.15.1 and RTA 1.13.48.0. In this RNA sequencing, library preparation and RNA isolation were performed by Illumina's simplified sample prep kits. Through this RNA sequencing process, we acquired FASTQ formatted output files for six independent sample libraries for each time point (*i.e.*, 3 dpi, 6 dpi, and 9 dpi), hence 36 libraries in total.


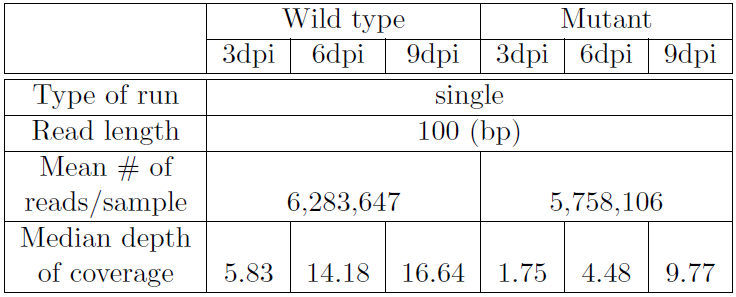


However, we only used sequencing datasets of the last two time points (6 dpi and 9 dpi) to specifically consider gene regulatory mechanism of the latter stages of stalk rot pathogenesis. The sequencing reads of 24 libraries were aligned to the reference genome of *F. verticillioides* strain 7600 40 ^1^ obtained from Broad Institute (http://www.broadinstitute.org). To acquire read counts of all *F. verticillioides* genes, we performed alignment by Bowtie2 ^2^, known to be better for relatively longer reads and gapped alignment, along with a subsequent process using a NGS analysis tool called Samtools ^3^. In our filtering process, we filtered genes that were not expressed in more than half of the replicates. However, we did not discard genes that expressed in at least 70% of the replicates in one strain even when these were not expressed at all in the other strain. This was to ensure that we keep genes that are extremely differentially expressed in different sample types (*i.e*., wild type vs Δfsr1 mutant) for our downstream analysis. After filtering Subsequently, 9,446 genes remained for our normalization and downstream analyses. Normalization of the read counts by each gene length was completed to obtain relative expression levels for each gene. Subsequent normalization was also performed across all replicates based on the expression level of two *β*-tubulin genes (FVEG_05512 and FVEG_04081) since they are often utilized for normalization of *F. verticillioides* and are considered as representative housekeeping genes showing exceptional constitutive expression in many fungal species. In this normalization, the mean expression levels of the *β*-tubulin genes were utilized as criteria.

For the purpose of co-expression network construction, we estimate the partial correlation between *F. verticillioides* genes. Partial correlation measures the strength of association between two random variables (in this case, two gene expression levels), in a way that removes the effect of other control random variables (in this case, other genes of *F. verticillioides*). Computationally, this can be done by estimating the covariance matrix followed by inversion of the matrix. Estimation of the covariance matrix is not affected by any affine transformation, hence the normalization techniques that are linear in nature do not affect the estimation results. As a result, we would like to emphasize that the choice of normalization technique has little (if any) impact on the co-expression network construction. The main reason we have normalized the gene expression levels based on the housekeeping genes was to make the gene expression levels easier to interpret by showing how a given gene is expressed relative to house-keeping genes that may not be directly related to the pathogenicity of *F. verticillioides*.

In order to support this, we performed comparison analysis of co-expression networks that were constructed based on datasets normalized by different combination of methods (within replicates & between replicates). We applied four different combinations of normalization such as TPM-TMM, TPM-RLE, RPKM-TMM, RPKM-control, where TMM is edgeR normalization method, RLE is DESeq2 normalization method, and beta-tubulin was used for the control. We inferred three different co-expression networks with three distinct sizes using partial correlation for each dataset (normalized by each combination). We finally compared those networks built based on different normalization methods by counting number of common edges. The percentages of common edges between networks are shown in the following Table.

| Network size  (# of edge) | TPM-TMM ↔  TPM-RLE | TPM-TMM ↔ RPKM-TMM | RPKM-control ↔ RPKM-TMM |
| --- | --- | --- | --- |
| ≈ 200,000 | 86% | 90% | 73% |
| ≈ 600,000 | 90% | 92% | 79% |
| ≈ 1,000,000 | 93% | 93% | 83% |

According to the Table, most of the percentages of edges in common between networks constructed in different conditions are higher than 80%, and the percentages can possibly be even higher in bigger network sizes. Therefore, please note that selection of normalization method does not have significant effect on our approach.

The general procedure for inferring functional subnetwork modules was described in our earlier publication, but a few factors were updated in this study. In this Supplementary Materials and Methods, we provide additional detail to our methodology used in this study. Here, we utilized only two time points (6 dpi & 9 dpi) for the analysis. Partial correlation cutoff values of the co-expression networks were different, when compared to our earlier study , due to the adjustment of network sizes. Based on the preprocessed gene expression levels, we constructed five different co-expression networks with five distinct partial correlation cutoff levels to reduce the dependency on certain cutoff values. We also converted the gene expression data into log likelihood ratio (LLR) matrix to analyze the expression probabilistically for subsequent analysis. Next, we searched for the most significantly differentially expressed genes (*i.e.*, top 1%) between the two fungal strains (wild type vs Δfsr1 mutant) as seed genes, *i.e.* the first member genes of subnetwork modules.

Equipped with the seed genes and the co-expression networks, we started to search for subnetwork modules associated with the *F. verticillioides* pathogenicity. For each seed gene, we added one of the connected genes to the seed gene and computed probabilistic activity level of each module, and also measured the difference level of the module using *t*-test statistics score between the two fungal strains ^4^. We selected up to three subnetwork modules whose difference of subnetwork activity levels between the two fungal strains (wild type vs Δfsr1 mutant) is either the optimum or suboptimum with certain conditions: i) discriminative power enhance (estimated by *t*-test statistics) of extended subnetwork module should be at least 10%, ii) discriminative power enhance gap between the optimum and the suboptimal should be less than 2%. For each module, we consistently expanded it by appending one of connected genes to any module members utilizing this computationally efficient branching-out technique until it did not satisfy minimum discriminative power increase any more. The entire searching process was reiterated for every seed genes and for the five co-expression networks. We probabilistically searched for subnetwork modules whose member genes have high likelihood of associated expression patterns to each other over all the replicates using the LLR matrix, whose values at each row allow us to predict how likely each gene is associated with either the wild type or the Δfsr1 mutant. As a result, our network-based computational analysis approach found candidate subnetwork modules that show a harmonious coordination between member genes as well as an association with pathogenicity by demonstrating strong differential activity between the two strains (*i.e.*, wild type vs Δfsr1 mutant).

In order to compute the pathogenicity-associated strength of a potential subnetwork, as described in the previous paragraph, we needed a method to infer the subnetwork activity level based on expression levels of the member genes. In this probabilistic inference, we adopted the probabilistic pathway activity inference scheme ^5^ to probabilistically predict the subnetwork activity level. Suppose we have $\boldsymbol{\Omega}\boldsymbol{=\{}\boldsymbol{g}_{\boldsymbol{1}}\boldsymbol{,}\boldsymbol{g}_{\boldsymbol{2}}\boldsymbol{,}\boldsymbol{g}_{\boldsymbol{3}}\boldsymbol{,}\boldsymbol{\ldots}\boldsymbol{,}\boldsymbol{g}_{\boldsymbol{n}}\boldsymbol{\}}$**,** a set of genes that belong to a subnetwork module of interest, and $\boldsymbol{e=\{}\boldsymbol{e}^{\boldsymbol{1}}\boldsymbol{,}\boldsymbol{e}^{\boldsymbol{2}}\boldsymbol{,}\boldsymbol{e}^{\boldsymbol{3}}\boldsymbol{,}\boldsymbol{\ldots}\boldsymbol{,}\boldsymbol{e}^{\boldsymbol{n}}\boldsymbol{\}}$**,** expression levels of the member genes in a given subnetwork module. The subnetwork module activity level can be measured by

$$\boldsymbol{\delta}\left( \boldsymbol{e} \right)\boldsymbol{=}\sum_{\boldsymbol{k=1}}^{\boldsymbol{n}} \boldsymbol{\sigma}^{\boldsymbol{k}}\boldsymbol{(}\boldsymbol{e}^{\boldsymbol{k}}\boldsymbol{)}$$

 (Equation S1)

where $\boldsymbol{\sigma}^{\boldsymbol{k}}\boldsymbol{(}\boldsymbol{e}^{\boldsymbol{k}}\boldsymbol{)}$ is the LLR between the two strains (*i.e.*, wild type vs. Δfsr1 mutant) defined as follows

$$\boldsymbol{\sigma}^{\boldsymbol{k}}\left( \boldsymbol{e}^{\boldsymbol{k}} \right)\boldsymbol{=log}\mathbf{}\boldsymbol{[}\frac{\boldsymbol{f}_{\boldsymbol{1}}^{\boldsymbol{k}}\left( \boldsymbol{e}^{\boldsymbol{k}} \right)}{\boldsymbol{f}_{\boldsymbol{2}}^{\boldsymbol{k}}\left( \boldsymbol{e}^{\boldsymbol{k}} \right)}\boldsymbol{]}$$

(Equation S2)

where $\boldsymbol{f}_{\boldsymbol{1}}^{\boldsymbol{k}}\boldsymbol{(e)}$ is the conditional probability density function (PDF) of the expression level of gene $g_{k}$ in wild type and $\boldsymbol{f}_{\boldsymbol{2}}^{\boldsymbol{k}}\boldsymbol{(e)}$ is the conditional PDF of the expression level of gene $g_{k}$ in the mutant. With this probabilistic quantification, we can compute the activity level $\boldsymbol{\delta(e)}$ of the given subnetwork module based on the expression levels of the member genes. It is important to note that the concept of this probabilistic approach came from naive Bayes model. We can also further evaluate the discriminative power of the module between the two conditions based on *t*-test statistics as follows

$$\boldsymbol{t}\left( \boldsymbol{\Omega} \right)\boldsymbol{=}\frac{\boldsymbol{\mu}_{\boldsymbol{1}}\boldsymbol{-}\boldsymbol{\mu}_{\boldsymbol{2}}}{\sqrt{\frac{\boldsymbol{s}_{\boldsymbol{1}}^{\boldsymbol{2}}}{\boldsymbol{n}_{\boldsymbol{1}}}\boldsymbol{+}\frac{\boldsymbol{s}_{\boldsymbol{2}}^{\boldsymbol{2}}}{\boldsymbol{n}_{\boldsymbol{2}}}}}$$

(Equation S3)

where $\mu_{1}$ and $s_{1}^{2}$ are the mean and the variance of the subnetwork module activity level in wild type, and $\mu_{2}$ and $s_{2}^{2}$ are the mean and the variance of the activity level in the Δfsr1 mutant. $n_{1}$ and $n_{2}$ are the number of replicates in the respective conditions.

1. **Supplementary Figure**

**Fig. S1.** (A) The targeted gene disruption for *FvSYN1* and *FvEBP1* was achieved by homologous recombination with following our standard split marker strategy ^4^. Schematic depiction of wild type (WT) *FvSYN1* and *FvEBP1* loci before and after homologous recombination are shown. Primers used in these experiments are listed in Table S4. Disruption constructs were generated by single Joint-PCR, and Hygromycin phosphotransferase (*HYG*) was used as the selective marker. Probe used for Southern analysis is shown in gray bar. (B) The gene deletion was confirmed by Southern analysis. Anticipated band sizes before and after recombination are indicated on the left. Strain with asterisk were selected for further experiments.

**3. Supplementary Tables**

**Table S1. Heat map genes (separate xlsx file)**

**Table S2. Summary of genes in *F. verticillioides* subnetwork modules in Fig. 4 (separate xlsx file)**

**Table S3. Summary of genes in maize subnetwork modules in Fig. 7 (separate xlsx file)**

**Table S4. Primers used in this study. The underlined sequences were for fusion purpose.**

| **Primer Name** | **5’-3’ sequence** |
| --- | --- |
| 1F | ACC AGG ATT ATT GGA GAG CAG G |
| 2R | TAG ATG CCG ACC GGG AAC TGA GCT GGA GCT CTG CTT TG |
| 3F | CCA CTA GCT CCA GCC AAG ACG ATG AGA GAA TGG TTC GGA G |
| 4R | TTC AAT CCT TGC AGC TGG TG |
| 5F | CAT ACT GAC ATG GCT TGA GG |
| 6R | CTT CGC TTC TTG TTG TCG TT |
| 7R | ACG CAA TTC ACG ACC GCC TAG |
| 8F | TAG AGA GCT ATG GAC AAG CTG G |
| 9R | TAG ATG CCG ACC GGG AAC GTC AGT ATC TGC TGT GCA AGA G |
| 10F | CCA CTA GCT CCA GCC AAG AGG ACA GGT GAC AGA CAA GTT G |
| 11R | TAA TGG CAG CAA CAC TCA CG |
| 12F | ACG CTG TTC GAT GTG TTT CGC |
| 13R | ACC AAG ACT GGC GAA GAA GAG C |
| 14F | ATC GTG GAA ACG CCA GAC TG |
| 3416-F | GGCCGTGGTCGGATCAATACTA |
| 3416-R | TGTCTCGGCGATGTGAGAGATT |
| 4212-F | ATGGCCTCGCAAGCAGATTACA |
| 4212-R | TCAGTCTTGCCATCAACAACGAGAA |
| 8818-F | AAGA TCC TGT CCT GAA GGA AGT TGG |
| 8818-R | CTTCGCCATAAGGACCTGGGTTTC |
| 9111-F | TAAGGATCGACCGAGTGACGAAATG |
| 9111-R | ACGTAGGATTGGACCCGTTGAAA |
| 3392-F | CCGATGGCTAGGAGTGTCGATAG |
| 3392-R | TGGTAGGAGTGTCTCAGCAAGG |
| 4259-F | CCATGTGTTTCCAGCGCAATCT |
| 4259-R | TCG ATG AGG CAA CTT CGT CCA T |
| 9144-F | CACCTTCAGCACCAGAACCTACTTC |
| 9144-R | ACACTTCACACCGCCAGTACATG |
| 13321-F | CGGACACCGTGGAAGCATAGAT |
| 13321-R | ATGTCGTGCAGCCGTCTGATAA |

**References**

1 Ma, L. J. *et al.* Comparative genomics reveals mobile pathogenicity chromosomes in *Fusarium*. *Nature* **464**, 367-373 (2010).

2 Langmead, B. & Salzberg, S. L. Fast gapped-read alignment with Bowtie 2. *Nature Methods* **9**, 357-359 (2012).

3 Li, H. *et al.* The Sequence Alignment/Map format and SAMtools. *Bioinformatics* **25**, 2078-2079 (2009).

4 Kim, M., Zhang, H., Woloshuk, C. P., Shim, W. B. & Yoon, B.-J. Computational prediction of pathogenic network modules in *Fusarium verticillioides*. *IEEE/ACM Transactions on Computational Biology and Bioinformatics* **15**, 506-515 (2018).

5 Su, J. J., Yoon, B. J. & Dougherty, E. R. Accurate and Reliable Cancer Classification Based on Probabilistic Inference of Pathway Activity. *PLoS One* **4**, e8161 (2009).
